# The development of nomograms to predict multiple pregnancy and multiple birth in frozen- thawed embryo transfer cycles: a retrospective study

**DOI:** 10.3389/fendo.2026.1930195

**Published:** 2026-09-25

**Authors:** Qi Cao

**Affiliations:** Department of Assisted Reproductive Medicine, Sichuan Provincial People’s Hospital, University of Electronic Science and Technology of China, Chengdu, China

**Keywords:** assisted reproductive technology, frozen-thawed, multiple birth, multiple pregnancy, nomogram, prediction model

## Abstract

**Objective:**

This study aimed to identify key factors influencing multiple pregnancy (MP) and multiple birth (MB) rates in frozen- thawed embryo transfer (FET) cycles, and to develop and validate clinically practical prediction nomograms for MP, MB, clinical pregnancy (CP), and live birth (LB).

**Methods:**

A total of 8,687 FET cycles performed between January 2014 and January 2024 were retrospectively analyzed. Cycles were randomly divided into a training set (n=6,081) and a validation set (n=2,606). Lasso regression was applied to screen independent predictive factors for MP, MB, CP, and LB. Nomogram models were constructed, and their performance was evaluated using the area under the receiver operating characteristic curve (AUC), calibration curves, decision curve analysis (DCA), and internal validation with bootstrapping and random splitting.

**Results:**

Seven predictors were identified for MP and MB: female age, male age, endometrial thickness on the transformation day, number of transferred embryos, number of transferred high- quality embryos, number of transferred blastocysts, and total number of remaining embryos (including cleavage-stage and blastocyst embryos of all quality grades) after transfer. For CP, predictors included female age, AMH, endometrial thickness on the transformation day, number of transferred embryos, number of transferred high-quality embryos, number of transferred blastocysts, and remaining embryos; LB predictors further included male age. The AUCs of the MP nomogram were 0.798 (training) and 0.804 (validation); those of the MB nomogram were 0.800 and 0.809, respectively. Calibration curves were close to the ideal diagonal, and DCA confirmed clinical net benefit within threshold probabilities of 1%–48% for MP and 1%–60% for MB. The CP and LB models showed moderate discriminative ability with AUCs around 0.69.

**Conclusion:**

We developed and internally validated four nomograms incorporating routine clinical and embryological indicators to estimate individual risks of MP, MB, CP and LB before FET. These tools enable personalized pre-transfer risk assessment and can serve as auxiliary references for clinicians’ patient counselling. External multicenter validation and prospective trials are required to confirm their clinical value before broad routine application.

## Introduction

Multiple pregnancy remains a major complication of assisted reproductive technology (ART) worldwide (1, 2). The global incidence of multiple pregnancy after ART ranges from 2% to 35%, and the rate in FET cycles is approximately 13%, markedly higher than the 1.0%–1.2% observed in natural conception (3, 4). Multiple pregnancy is strongly associated with elevated risks of preterm birth, low birth weight, cerebral palsy, neurodevelopmental disorders, and perinatal mortality (5, 6). Iatrogenic multiple pregnancies cause substantial physical and psychological distress for patients and impose a considerable economic burden on healthcare systems (7, 8).

To mitigate multiple birth rates (MBR), the American Society for Reproductive Medicine (ASRM) recommends elective single embryo transfer (eSET) in patients aged ≤37 years (9). In 2018, the Chinese Expert Consensus on the Number of Embryos Transferred advocated eSET in the first transfer cycle and a maximum of two embryos per transfer cycle regardless of age (10). However, the adoption of eSET remains limited in many clinical settings, and clinicians face the persistent challenge of lowering MBR without compromising live birth rates (LBR) (11, 12).

Accurate predictive tools are urgently needed to balance pregnancy success and multiple pregnancy risk (13, 14). Although numerous prediction models for ART outcomes have been developed, most focus on fresh embryo transfer or pooled fresh and FET cycles, and few are specifically designed and validated for MP and MB in FET cycles (15, 16). This study aimed to develop and internally validate prediction nomograms for MP, MB, CP, and LB following FET using a large single-center cohort, to support personalized clinical decision-making.

## Materials and methods

### Study design and participants

This retrospective study collected all frozen-thawed embryo transfer (FET) cycles performed at the Department of Assisted Reproductive Medicine, Sichuan Provincial People’s Hospital between January 2014 and January 2024 as the initial candidate pool. Cycles were sequentially excluded if they met any of the predefined exclusion criteria: donor sperm cycles, donor oocyte cycles, chromosomal abnormalities in either partner, preimplantation genetic testing (PGT) cycles, and cycles with missing key data. After stepwise screening and removal of ineligible records, a total of 8,687 valid FET cycles were ultimately retained for analysis. Among these cycles, 4,876 resulted in clinical pregnancies, within which 1,502 were multiple pregnancies; 4,007 cycles achieved live birth, and 1,102 of these live-birth cycles corresponded to multiple births. The complete participant screening workflow is visually summarized in **Figure 1**. The study was approved by the Hospital Ethics Committee (No.: 2026-420), and informed consent was waived given its retrospective design.

**Figure 1:**
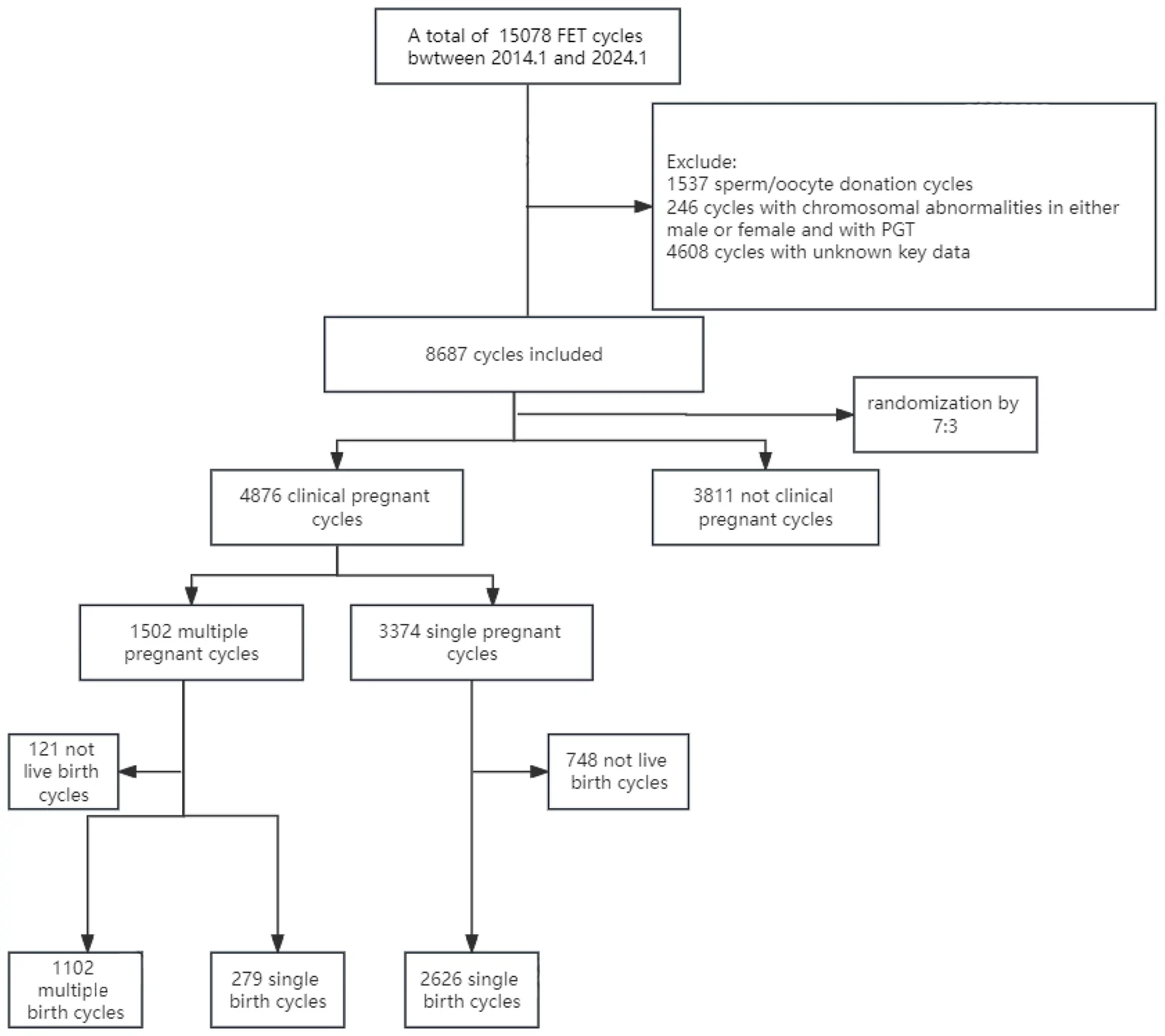
Study flow chart. Flow diagram of patient enrollment, exclusion criteria, grouping into training and validation sets, and outcomes included in the final analysis.

### Ovarian stimulation, insemination, and embryo culture

Controlled ovarian hyperstimulation protocols and gonadotropin dosages were individualized. Human chorionic gonadotropin (hCG) was administered when at least one follicle reached ≥18 mm. Oocyte retrieval was performed 36 hours later under transvaginal ultrasound guidance. Conventional IVF or ICSI was used according to semen parameters. Embryos were cultured in a single medium at 37 °C under 5% O_2_, 6% CO_2_, and balanced nitrogen. All supernumerary embryos, regardless of whether fresh-embryo transfer was performed, were cryopreserved by vitrification.

### Endometrial preparation and FET

Endometrial preparation included GnRH-agonist hormone replacement therapy (GnRHa-HRT), HRT, natural cycles, and ovulation induction cycles. Embryo transfer was performed under abdominal ultrasound guidance.

### Embryo and endometrial assessment

Cleavage-stage embryos of Grade I or II with uniform blastomeres and minimal fragmentation were defined as high-quality. Blastocysts graded ≥4BB by the Gardner system were considered high-quality (17). Endometrial thickness (EMT) was measured on the transformation day by transvaginal ultrasound, as the maximum anteroposterior diameter in the sagittal plane. All embryo-related predictors used in the models were uniformly operationalized according to routine clinical recording standards at our center. The number of transferred embryos referred to the total embryos placed into the uterine cavity per FET cycle; transferred high-quality embryos were counted based on standardized morphological grading as defined above; the number of transferred blastocysts referred to the count of blastocyst-stage embryos that were actually transferred after thawing; the total number of remaining embryos denoted all supernumerary cryopreserved cleavage-stage and blastocyst-stage embryos. All embryological data were prospectively documented in electronic medical records by laboratory embryologists, enabling consistent variable collection and reproducible model application in routine ART practice.

### Outcome definitions

- Clinical pregnancy (CP): Presence of a gestational sac with fetal heartbeat on ultrasound 4–5 weeks after transfer.
- Multiple pregnancy (MP): Two or more fetal heartbeats detected.
- Live birth (LB): At least one live neonate delivered at ≥28 weeks of gestation.This gestational cutoff follows the standard classification of viable preterm infants in Chinese clinical perinatal practice; infants born before 28 weeks are categorized as extremely preterm infants with markedly different survival and clinical management characteristics.
- Multiple birth (MB): At least two live neonates delivered at ≥28 weeks of gestation, with the same ≥28-week gestational cutoff rationale as live birth definition.

### Statistical analysis

Data were analyzed using R software (v2.1.1). Continuous variables were expressed as mean ± SD or median (IQR); categorical variables as counts (percentages). Groups were compared using t-tests, Kruskal-Wallis tests, or chi-square tests. A two-sided P<0.05 was considered statistically significant.

Cycles were randomly split into training (70%) and validation (30%) sets. Lasso regression was used for variable selection (**Figure 2**). Prior to model building, multicollinearity was assessed using GVIF^(1/(2*DF)). All values were below 2.5, and no predictors were excluded due to collinearity concerns. Nomograms were developed and evaluated for discrimination (AUC), calibration (calibration curves with bootstrapping), and clinical utility via decision curve analysis (DCA) (18, 19). To counteract the optimistic bias inherent to single random-split validation, internal bootstrapping with 500 resamples was performed to generate optimism-corrected AUC and calibration estimates.

**Figure 2:**
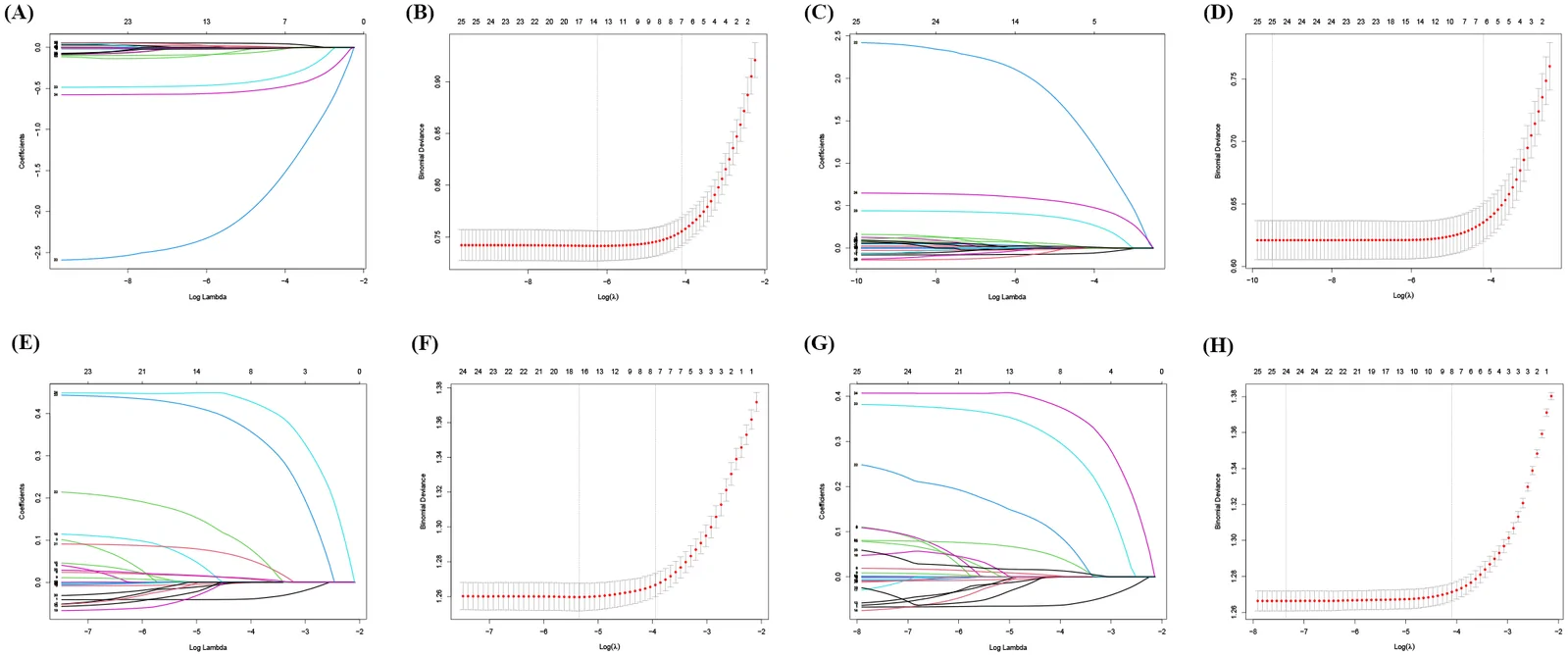
Variable selection for prediction models using Lasso regression. **(A, C, E, G)** Lasso coefficient profiles of the candidate features; **(B, D, F, H)** the selection of optimal parameters (lambda) by tenfold cross-validation, in which dotted vertical lines were drawn at the optimal values by using the minimum criteria and limits defined by 1 standard deviation. **(A, B)** for multiple pregnancy (MP) models; **(C, D)** for multiple birth (MB) models; **(E, F)** for clinical pregnancy (CP) models; **(G, H)** for live birth (LB) models.

## Results

### Baseline characteristics

A total of 8,687 frozen-thawed embryo transfer (FET) cycles were enrolled in this retrospective study, including 6,081 cycles in the training set and 2,606 cycles in the validation set. The overall clinical pregnancy rate was 56.1% (4,876/8,687), multiple pregnancy rate was 17.3% (1,502/8,687), live birth rate was 46.1% (4,007/8,687), and multiple birth rate was 12.7% (1,102/8,687). Baseline demographic, infertility, embryological, and endometrial preparation variables were well balanced between the training and validation cohorts, with no statistically significant differences (all P>0.05; **Table 1**).

**Table 1:** Baseline characteristics and clinical outcomes of the total cohort, training set, and validation set.

Variables	Total (n = 8687)	Training set (n = 6081)	Validation set (n = 2606)	P-value
Clinical pregnancy				0.934
No	3811 (43.9)	2666 (43.8)	1145 (43.9)	
Yes	4876 (56.1)	3415 (56.2)	1461 (56.1)	
Multiple pregnancy				0.25
No	7185 (82.7)	5011 (82.4)	2174 (83.4)	
Yes	1502 (17.3)	1070 (17.6)	432 (16.6)	
Live birth				0.998
No	4680 (53.9)	3276 (53.9)	1404 (53.9)	
Yes	4007 (46.1)	2805 (46.1)	1202 (46.1)	
Multiple birth				0.5
No	7585 (87.3)	5300 (87.2)	2285 (87.7)	
Yes	1102 (12.7)	781 (12.8)	321 (12.3)	
Female age (years)	32.4 ± 4.6	32.4 ± 4.6	32.4 ± 4.7	0.422
Male age (years)	34.5 ± 5.5	34.4 ± 5.4	34.6 ± 5.7	0.106
Infertility type				0.563
Primary	4208 (48.4)	2958 (48.6)	1250 (48)	
Secondary	4479 (51.6)	3123 (51.4)	1356 (52)	
Infertility factor				0.104
Tubal factor	4392 (50.6)	3108 (51.1)	1284 (49.3)	
Double factor	1721 (19.8)	1199 (19.7)	522 (20)	
Male factor	1549 (17.8)	1067 (17.5)	482 (18.5)	
Anovulation/PCOS	365 (4.2)	242 (4)	123 (4.7)	
Endometriosis	308 (3.5)	217 (3.6)	91 (3.5)	
Diminished ovarian reserve	179 (2.1)	125 (2.1)	54 (2.1)	
Unexplained	110 (1.3)	80 (1.3)	30 (1.2)	
Immune factor	25 (0.3)	12 (0.2)	13 (0.5)	
Uterine factor	38 (0.4)	31 (0.5)	7 (0.3)	
Duration of infertility (years)	3.0 (2.0, 5.0)	3.0 (2.0, 5.0)	3.0 (2.0, 5.0)	0.15
Number of prior premature births				0.051
0	8667 (99.8)	6071 (99.8)	2596 (99.6)	
1	20 (0.2)	10 (0.2)	10 (0.4)	
Number of prior induced abortions				0.137
0	8450 (97.3)	5928 (97.5)	2522 (96.8)	
1	224 (2.6)	145 (2.4)	79 (3)	
2	13 (0.1)	8 (0.1)	5 (0.2)	
Number of prior spontaneous abortions				0.651
0	7377 (84.9)	5166 (85)	2211 (84.8)	
1	996 (11.5)	698 (11.5)	298 (11.4)	
2	229 (2.6)	153 (2.5)	76 (2.9)	
3	66 (0.8)	51 (0.8)	15 (0.6)	
≥0	19 (0.2)	13 (0.2)	6 (0.3)	
Number of prior artificial abortions				0.338
0	6367 (73.3)	4475 (73.6)	1892 (72.6)	
1	1604 (18.5)	1115 (18.3)	489 (18.8)	
2	487 (5.6)	324 (5.3)	163 (6.3)	
3	181 (2.1)	132 (2.2)	49 (1.9)	
≥1	48 (0.5)	35 (0.6)	13 (0.4)	
Number of prior medical abortions				0.233
0	8277 (95.3)	5793 (95.3)	2484 (95.3)	
1	322 (3.7)	228 (3.7)	94 (3.6)	
2	61 (0.7)	39 (0.6)	22 (0.8)	
3	14 (0.2)	13 (0.2)	1 (0)	
≥0	13 (0.1)	8 (0.1)	5 (0.2)	
Number of prior ectopic pregnancies				0.734
0	6870 (79.1)	4799 (78.9)	2071 (79.5)	
1	1325 (15.3)	937 (15.4)	388 (14.9)	
2	403 (4.6)	285 (4.7)	118 (4.5)	
3	76 (0.9)	52 (0.9)	24 (0.9)	
≥0	13 (0.1)	8 (0.1)	5 (0.2)	
Number of prior pregnancies				
0	4213(48.5)	2962(48.7)	1251(48)	0.693
1	2089(24.0)	1470(24.2)	619(23.8)	
2	1247(14.4)	862(14.2)	385(14.8)	
3	597(6.9)	417(6.9)	180(6.9)	
≥.	541(6.2)	370(6.0)	171(6.5)	
Number of prior full term births				0.47
0	8199 (94.4)	5755 (94.6)	2444 (93.8)	
1	418 (4.8)	281 (4.6)	137 (5.3)	
2	58 (0.7)	37 (0.6)	21 (0.8)	
3	11 (0.1)	7 (0.1)	4 (0.2)	
≥0	1 (0.0)	1 (0)	0 (0)	
AMH(ng/ml)	3.8 (2.2, 6.4)	3.8 (2.2, 6.4)	3.9 (2.1, 6.4)	0.289
BMI(kg/m^2^)	22.0 ± 3.2	22.0 ± 3.1	22.1 ± 3.2	0.556
Sperm density (×10^6^/ml)	42.0 (24.2, 67.8)	42.0 (24.0, 68.0)	42.0 (25.0, 67.0)	1
Sperm normal morphology rate	2.5 (1.0, 3.9)	2.5 (1.0, 3.9)	2.5 (1.0, 3.9)	0.52
Non-progressive sperm motility rate	7.6 (5.0, 15.0)	7.6 (5.0, 15.2)	7.6 (5.0, 13.9)	0.329
Progressive sperm motility rate	43.8 (30.9, 55.6)	43.8 (31.1, 55.8)	43.7 (30.7, 55.3)	0.858
Number of oocyte retrieval cycles				0.849
1	7531 (86.7)	5272 (86.7)	2259 (86.7)	
2	916 (10.5)	640 (10.5)	276 (10.6)	
3	165 (1.9)	113 (1.9)	52 (2)	
≥2	75 (0.9)	56 (0.9)	19 (0.7)	
Ovarian stimulation protocols				0.675
Agonist protocol	5291 (60.9)	3720 (61.2)	1571 (60.3)	
Antagonist protocol	3042 (35.0)	2119 (34.8)	923 (35.4)	
Mild stimulation protocol	241 (2.8)	169 (2.8)	72 (2.8)	
PPOS protocol	110 (1.3)	71 (1.2)	39 (1.5)	
Natural cycle protocol	3 (0.0)	2 (0)	1 (0)	
Gonadotrophin dosage (IU)	2496.7 ± 966.1	2491.6 ± 971.7	2508.7 ± 953.1	0.451
Gonadotrophin duration (days)	11.9 ± 2.8	11.9 ± 2.8	12.0 ± 2.7	0.281
Insemination method				0.895
IVF	7156 (82.4)	5020 (82.6)	2136 (82)	
ICSI	1309 (15.1)	907 (14.9)	402 (15.4)	
TESA/PESA+ICSI	148 (1.7)	104 (1.7)	44 (1.7)	
IVF+ICSI/RICSI	74 (0.9)	50 (0.8)	24 (0.9)	
Number of oocytes collected	15.6 ± 7.5	15.6 ± 7.5	15.6 ± 7.6	0.721
Number of MII oocytes	13.4 ± 6.7	13.4 ± 6.7	13.4 ± 6.8	0.791
Number of fertilized oocytes	11.8 ± 6.3	11.8 ± 6.3	11.8 ± 6.3	0.999
Number of normally fertilized oocytes	8.9 ± 5.3	8.9 ± 5.3	9.0 ± 5.3	0.487
Number of cleavage-stage embryos	9.9 ± 5.3	9.9 ± 5.3	9.9 ± 5.3	0.62
Number of blastocytes cultured	8.0 (4.0, 11.0)	8.0 (4.0, 11.0)	8.0 (4.0, 12.0)	0.512
Number of day 3 (D3) frozen embyos	2.0 (0.0, 2.0)	2.0 (0.0, 2.0)	2.0 (0.0, 2.0)	0.904
Number of cryopreserved blastocysts	3.0 (1.0, 5.0)	3.0 (1.0, 5.0)	3.0 (1.0, 5.0)	0.353
Number of frozen embryos	5.1 ± 3.3	5.0 ± 3.3	5.1 ± 3.3	0.596
Ovulation induction outcome				0.702
Not transferred	7436 (85.6)	5213 (85.7)	2223 (85.3)	
Non-pregnancy	767 (8.8)	529 (8.7)	238 (9.1)	
Biochemical pregnancy	267 (3.1)	192 (3.2)	75 (2.9)	
Clinical pregnancy	217 (2.5)	147 (2.4)	70 (2.7)	
Number of transfer cycles				0.915
1	5651 (65.1)	3980 (65.4)	1671 (64.1)	
2	2392 (27.5)	1655 (27.2)	737 (28.3)	
3	528 (6.1)	367 (6)	161 (6.2)	
≥6	116 (1.3)	79 (1.3)	37 (1.4)	
FET protocols				0.203
GnRHa+HRT	5079 (58.5)	3581 (58.9)	1498 (57.5)	
HRT	3196 (36.8)	2227 (36.6)	969 (37.2)	
NC(natural regime)	280 (3.2)	181 (3)	99 (3.8)	
Ovulation induction	132 (1.5)	92 (1.5)	40 (1.5)	
Endometrial thickness on the transformation day(mm)	9.7 ± 1.9	9.7 ± 1.9	9.7 ± 1.9	0.928
Number of transferred embryos				0.606
1	2633 (30.3)	1859 (30.6)	774 (29.7)	
2	6019 (69.3)	4196 (69)	1823 (70)	
3	35 (0.4)	26 (0.4)	9 (0.3)	
Number of transferred high-quality embryos				0.094
0	4392 (50.6)	3028 (49.8)	1364 (52.3)	
1	3508 (40.4)	2493 (41)	1015 (38.9)	
2	787 (9.1)	560 (9.2)	227 (8.7)	
Number of transferred blastocysts				0.697
0	2296 (26.4)	1611 (26.5)	685 (26.3)	
1	2338 (26.9)	1654 (27.2)	684 (26.2)	
2	4052 (46.6)	2815 (46.3)	1237 (47.5)	
3	1 (0.0)	1 (0)	0 (0)	
Number of remaining embryos after transfer	2.0 (0.0, 4.0)	2.0 (0.0, 4.0)	2.0 (0.0, 4.0)	0.945

Continuous variables are presented as mean ± standard deviation or median (interquartile range, IQR). Categorical variables are presented as number (percentage). Differences between the training and validation sets were analyzed using independent t-tests, Kruskal–Wallis tests, or chi-square tests, as appropriate. A P-value < 0.05 was considered statistically significant.

The mean female age was 32.4 ± 4.6 years, mean male age was 34.5 ± 5.5 years, and mean body mass index (BMI) was 22.0 ± 3.2 kg/m². The median AMH level was 3.8 (2.2, 6.4) ng/ml, and median endometrial thickness on the transformation day was 9.7 ± 1.9 mm. Primary infertility accounted for 48.4% of cases, and secondary infertility for 51.6%. Tubal factor was the most common cause of infertility (50.6%), followed by double factors (19.8%) and male factors (17.8%). The majority of patients underwent 1 oocyte retrieval cycle (86.7%) and 1–2 FET cycles (92.6%). GnRHa-HRT was the most frequently used endometrial preparation protocol (58.5%), followed by conventional HRT (36.8%). Most cycles involved transfer of 2 embryos (69.3%), and 46.6% of cycles included transfer of 2 blastocysts. No significant intergroup differences were observed in sperm parameters, gonadotropin dosage and duration, insemination method, number of oocytes retrieved, number of frozen embryos, or number of remaining embryos after transfer (all P>0.05; **Table 1**). These data confirmed the homogeneity between the training and validation sets, providing a reliable foundation for subsequent model construction and validation.

### Predictive factors identified

Lasso regression identified seven independent predictors for MP and MB: female age, male age, EMT on transformation day, number of transferred embryos, number of transferred high-quality embryos, number of transferred blastocysts, and total number of remaining embryos after transfer (**Figures 2A–D**). For CP, predictors were female age, AMH, EMT, number of transferred embryos, number of transferred high-quality embryos, number of transferred blastocysts, and total number of remaining embryos after transfer (**Figures 2E, F**). LB predictors further included male age (**Figures 2G, H**).Multicollinearity testing via GVIF^(1/(2*DF)) yielded all values < 2.5, showing no problematic collinearity among predictors.

### Nomogram development

Based on the screened predictors, four visualized prediction nomograms were constructed for MP, MB, CP, and LB, respectively (**Figure 3**). Each predictor was assigned a weighted score according to its regression coefficient, and the total score was mapped to the corresponding predicted probability on the bottom scale of the nomogram.

**Figure 3:**
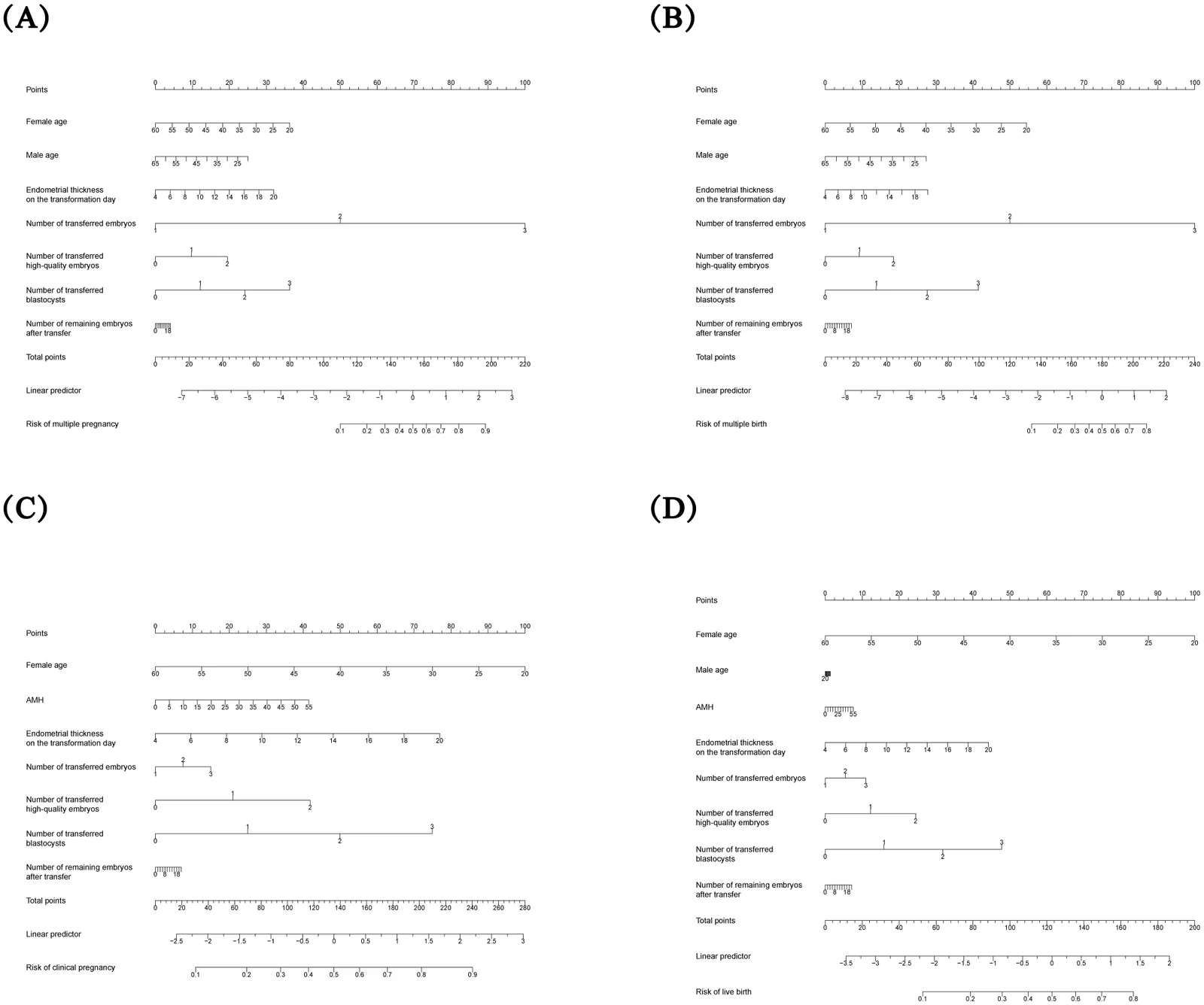
Developed nomograms for predicting **(A)** multiple pregnancy, **(B)** multiple birth, **(C)** clinical pregnancy and **(D)** live birth in frozen-thawed embryo transfer (FET) cycles. To use the nomogram, locate the value of each variable on its corresponding axis, draw a vertical line to the points axis to determine the score for each variable, sum all scores to obtain the total points, and draw a vertical line from the total points axis to the bottom probability axis to obtain the individual predicted probability.

To facilitate clinical application, web-based calculators embedded with complete regression formulas of the four nomograms were developed for rapid individualized risk assessment before embryo transfer (**Supplementary Figure S1**). In a typical illustrative case: female age 33 years, male age 36 years, AMH 4.0 ng/ml, endometrial thickness 8 mm, 2 transferred blastocysts including 1 high-quality embryo, and 2 remaining embryos after transfer. The predicted probabilities were calculated as: clinical pregnancy ≈67.17%, multiple pregnancy ≈26.60%, live birth ≈55.23%, and multiple birth ≈18.65%. This example demonstrated that the nomograms could intuitively and quantitatively estimate individual probabilities before FET, supporting personalized clinical decision-making.

### Model performance

Model performance is illustrated in **Figure 4**. For the multiple pregnancy (MP) nomogram, the AUC was 0.798 (95%CI: 0.786–0.811) in the training set and 0.804 (95%CI: 0.785–0.824) in the validation set, with a sensitivity of 0.62 and specificity of 0.86. For the multiple birth (MB) nomogram, the AUC was 0.800 (95%CI: 0.786–0.814) in the training set and 0.809 (95%CI: 0.787–0.831) in the validation set, with a sensitivity of 0.85 and specificity of 0.63. Both models achieved good to excellent discriminative capacity, with AUC values greater than 0.80.

**Figure 4:**
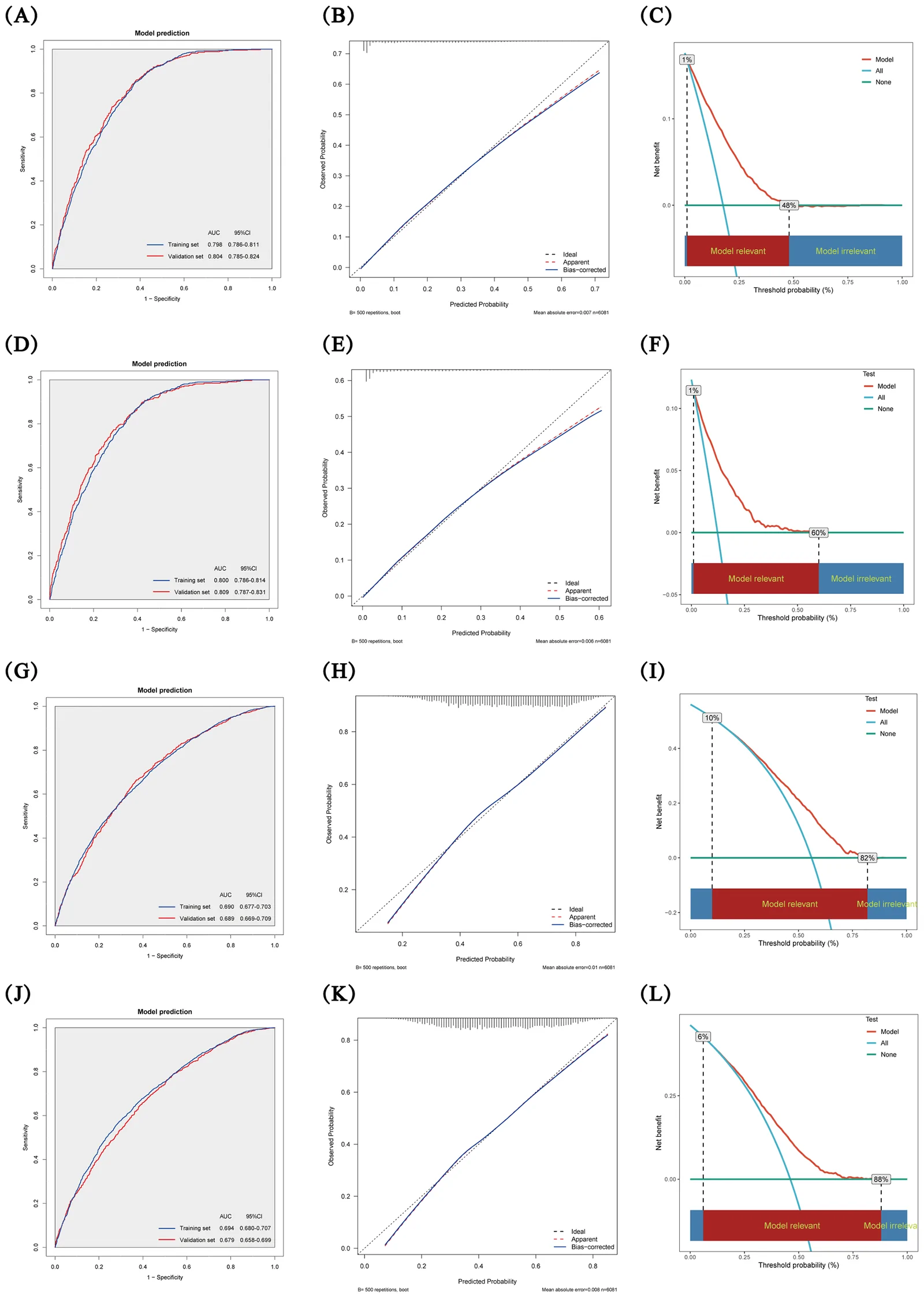
Performance evaluation of the prediction nomograms. **(A, D, G, J)** Receiver operating characteristic (ROC) curves; **(B, E, H, K)** Calibration curves; **(C, F, I, L)** Decision curve analysis (DCA). **(A–C)** for multiple pregnancy (MP) models; **(D–F)** for multiple birth (MB) models; **(G–I)** for clinical pregnancy (CP) models; **(J–L)** for live birth (LB) models.

Calibration curves showed excellent agreement between predicted probabilities and actual observed frequencies, closely approximating the ideal diagonal line, suggesting high consistency and reliability. Hosmer–Lemeshow goodness-of-fit tests confirmed no significant deviation from perfect calibration (MP: P = 0.28; MB: P = 0.178). Decision curve analysis (DCA) revealed that the MP nomogram provided positive net benefit within threshold probabilities ranging from 1% to 48%, and the MB nomogram within 1% to 60%, verifying favorable clinical applicability.

For the clinical pregnancy (CP) and live birth (LB) nomograms, the discriminative performance was moderate. The AUC of the CP model was 0.690 in the training set and 0.689 in the validation set. The AUC of the LB model was 0.694 in the training set and 0.679 in the validation set. Both models achieved acceptable discriminative ability and goodness-of-fit, consistent with most published prediction models for live birth following ART.

Internal validation via bootstrapping and random splitting further confirmed the stability and generalizability of all four models. Overall, the MP and MB models exhibited superior predictive performance, whereas the CP and LB models offered moderate-value references for individualized outcome estimation in clinical practice.

## Discussion

Determining the optimal number of embryos to transfer is one of the most complex decisions in IVF, requiring careful balancing between live birth and multiple pregnancy risks (13, 20). Our study developed and validated four prediction nomograms for FET cycles, with the MP and MB models showing good discrimination, calibration, and clinical utility. To our knowledge, this is one of the few large-scale studies focusing specifically on MP and MB prediction in pure FET cycles (15, 21).

Female age was consistently predictive of all outcomes, consistent with well-established evidence that ovarian reserve and embryo competence decline with age (22, 23). Female age is an independent risk factor for pregnancy outcomes in assisted reproductive technology, which further supports its essential role in pretreatment evaluation and prognostic stratification. With advancing female age, the quantity and quality of oocytes decrease progressively, leading to lower implantation potential and higher rates of pregnancy loss, which explains its stable predictive value across all endpoints in our models.

Male age also entered the final models, supporting emerging evidence that advanced paternal age adversely affects embryo development and pregnancy outcomes (24, 25). The identification of male age as a predictive factor underscores the growing recognition of paternal contributions to reproductive success. Advanced paternal age is associated with declined sperm quality, impaired embryo developmental competence, and elevated risk of early miscarriage, reinforcing the necessity of comprehensive couple-based fertility evaluation (25). These findings highlight the importance of assessing both partners for a holistic fertility evaluation, which can facilitate more targeted therapeutic strategies and optimize clinical outcomes in ART.

Endometrial thickness on the transformation day was an independent predictor, confirming that adequate endometrial development improves implantation (26, 27). Identifying the optimal range of endometrial thickness is critical for enhancing pregnancy outcomes in fertility treatment. A sufficiently thick and receptive endometrium provides a favorable microenvironment for embryo adhesion and invasion, and individualized endometrial preparation protocols may further improve implantation efficiency in FET cycles.

The number of transferred embryos, high-quality embryos, and blastocysts were strong predictors of multiple pregnancy, reflecting the high implantation potential of morphologically good blastocysts (17, 28). The number of high-quality embryos transferred emphasizes the pivotal role of embryo quality in achieving favorable clinical outcomes, which is consistent with previous prediction models in frozen-thawed embryo transfer cycles (29). Compared with cleavage-stage embryos, blastocysts undergo more rigorous natural selection during *in vitro* culture, which eliminates embryos with limited developmental potential. This enhanced selection process promotes better embryo-endometrium synchronization, thereby increasing implantation rates and shortening time to pregnancy.

The total number of remaining embryos may serve as a surrogate marker of overall embryo cohort quality and ovarian response. A higher number of remaining embryos reflects a more robust ovarian response and a larger pool of viable embryos with diverse developmental stages and quality grades, which is indirectly associated with improved implantation and pregnancy potential. Notably, we separately incorporated the number of transferred high-quality embryos and transferred blastocysts into the prediction models to adjust for embryo quality and developmental stage; the total remaining embryo count remains an independent complementary predictor that captures the cumulative embryo reserve of each patient.

Intriguingly, the MP and MB models achieved AUCs >0.80, outperforming CP and LB models (AUC≈0.69). This is consistent with previous reports that LB prediction remains challenging, with most models yielding AUC <0.70 (30, 31). This performance discrepancy is biologically plausible: multiple pregnancy risk is predominantly governed by clearly documented embryological indicators such as the number and quality of transferred embryos, which are fully captured in our database. In comparison, clinical pregnancy and live birth are affected by a wider range of uncollected variables including subtle endometrial immune status, individual lifestyle habits and psychological factors, leading to limited predictive accuracy of CP and LB nomograms.

For clinical application, we propose a hierarchical usage strategy for these four nomograms. The MP and MB nomograms with AUC >0.80 are the primary tool for stratifying multiple gestation risk, which addresses the core objective of the present study. The CP and LB models with moderate discriminative ability can only serve as auxiliary references rather than independent decision-making instruments. When clinicians decide the number of embryos to transfer, they should prioritize risk probabilities generated by MP/MB models and take CP/LB predicted results as secondary background information.

Our study has several notable strengths. It features a relatively large sample size and rigorous internal validation, with comprehensive evaluation covering discrimination, calibration, and clinical utility. Furthermore, the exclusive focus on FET cycles helps minimize confounding factors caused by heterogeneous fresh-cycle conditions, strengthening the homogeneity of the study population. These nomograms offer a quantitative, patient-specific tool to inform embryo-transfer decision-making, moving beyond purely empirical strategies. By identifying high-risk patients, clinicians may consider recommending eSET to reduce multiple-pregnancy risk while preserving acceptable live-birth rates, which aligns with global efforts to improve ART safety (9, 10, 12).

Limitations include its retrospective single-center design and the lack of external validation in independent cohorts, which may restrict its generalizability to populations with different patient characteristics, endometrial preparation regimens, and embryo laboratory protocols. Additionally, objective standardized scoring for embryo transfer difficulty was not routinely documented in electronic medical records and could not be incorporated into the present analysis, which might introduce residual prediction bias. In addition, live and multiple birth were defined using a gestational cutoff of 28 weeks according to local clinical practice, which may reduce direct comparability with ART studies adopting different gestational thresholds for live birth endpoints. Multi-level regression approaches for future multi-center prospective studies with external validation are warranted before widespread clinical adoption of these nomograms.

## Conclusion

We developed and internally validated four nomograms incorporating routine clinical and embryological indicators to estimate individual risks of MP, MB, CP and LB before FET. These tools enable personalized pre-transfer risk assessment and can serve as auxiliary references for clinicians’ patient counselling. External multicenter validation and prospective trials are required to confirm their clinical value before broad routine application.

## Data Availability

The original contributions presented in the study are included in the article/**Supplementary Material**. Further inquiries can be directed to the corresponding author.

## Supplementary material

Supplementary Figure 1 Web-based calculators embedded with complete regression formulas of the four nomograms. Regression coefficients of the nomogram models for multiple pregnancy **(A)**, multiple birth **(B)**, clinical pregnancy **(C)**, and live birth **(D)**. The figure includes intercept and regression weight for each independent predictor, which can be directly used to recalculate predicted risk probabilities for external cohorts.

## References

[1] Kim H Claffey AJ Doody KJ Doody KM . Mitigating multiples, less is more: examining trends in multifetal gestation rates with assisted reproductive technology. J Assisted Reprod Genet. (2024) 41:2301–10. doi: 10.1007/s10815-024-03208-4 39085741 PMC11405601

[2] Dietrich JE Cáceres Valcárcel I Capp E Strowitzki T Germeyer A . High multiple pregnancy rates after double embryo transfers in human: a retrospective cohort study. Reprod Fertility. (2025) 6:e240078. doi: 10.1530/raf-24-0078 40111385 PMC12002859

[3] Dyer S Chambers GM Jwa SC Baker VL Banker M de Mouzon J . International Committee for Monitoring Assisted Reproductive Technologies world report: assisted reproductive technology, 2019. Fertility Sterility. (2025) 124:679–93. doi: 10.1016/j.fertnstert.2025.06.003 40513636

[4] Kulkarni AD Jamieson DJ Jones HW Jr. Kissin DM Gallo MF Macaluso M . Fertility treatments and multiple births in the United States. N Engl J Med. (2013) 369:2218–25. doi: 10.1056/nejmoa1301467 24304051

[5] Marleen S Kodithuwakku W Nandasena R Mohideen S Allotey J Fernández-García S . Maternal and perinatal outcomes in twin pregnancies following assisted reproduction: a systematic review and meta-analysis involving 802 462 pregnancies. Hum Reprod Update. (2024) 30:309–22. doi: 10.1093/humupd/dmae002 38345641 PMC11063550

[6] American College of Obstetricians and Gynecologists . Multifetal gestations: Twin, triplet, and higher-order multifetal pregnancies: ACOG Practice Bulletin, Number 231. Obstetrics Gynecology. (2021) 137:e145–62. doi: 10.1097/aog.0000000000004397 34011891

[7] Wyns C Bergh C Calhaz-Jorge C De Geyter C Kupka MS Motrenko T . ART in Europe, 2016: results generated from European registries by ESHRE. Hum Reprod Open. (2020) 2020:hoaa032. doi: 10.1093/hropen/hoaa032 32760812 PMC7394132

[8] Practice Committee of Society for Assisted Reproductive Technology Practice Committee of American Society for Reproductive Medicine . Elective single-embryo transfer. Fertility Sterility. (2012) 97:835–42. doi: 10.1007/978-1-60327-392-3_11 22196716

[9] Practice Committee of the American Society for Reproductive Medicine and the Practice Committee for the Society for Assisted Reproductive Technologies . Guidance on the limits to the number of embryos to transfer: a committee opinion. Fertility Sterility. (2021) 116:651–4. doi: 10.1016/j.fertnstert.2021.06.050 34330423

[10] Chen H Wu S Su W Zhao J Wu Y . Comparison of pregnancy outcomes among patients of different ages who underwent frozen-thawed high-quality single blastocyst transfer. BMC Pregnancy Childbirth. (2024) 24:276. doi: 10.1186/s12884-024-06451-w 38622514 PMC11017700

[11] Ma S Peng Y Hu L Wang X Xiong Y Tang Y . Comparisons of benefits and risks of single embryo transfer versus double embryo transfer: a systematic review and meta-analysis. Reprod Biol Endocrinol RB&E. (2022) 20:20. doi: 10.1186/s12958-022-00899-1 35086551 PMC8793185

[12] Alteri A Arroyo G Baccino G Craciunas L De Geyter C Ebner T . ESHRE guideline: number of embryos to transfer during IVF/ICSI†. Hum Reprod (Oxford England). (2024) 39:647–57. doi: 10.1093/humrep/deae010 38364208 PMC10988112

[13] Awadalla MS Bendikson KA Ho JR McGinnis LK Ahmady A . A validated model for predicting live birth after embryo transfer. Sci Rep. (2021) 11:10800. doi: 10.1038/s41598-021-90254-y 34031492 PMC8144418

[14] Williams Z Banks E Bkassiny M Jayaweera SK Elias R Veeck L . Reducing multiples: a mathematical formula that accurately predicts rates of singletons, twins, and higher-order multiples in women undergoing *in vitro* fertilization. Fertility Sterility. (2012) 98:1474–80.e2. doi: 10.1016/j.fertnstert.2012.08.014 22985944

[15] Luke B Brown MB Wantman E Stern JE Baker VL Widra E . A prediction model for live birth and multiple births within the first three cycles of assisted reproductive technology. Fertility Sterility. (2014) 102:744–52. doi: 10.1016/j.fertnstert.2014.05.020 24934487 PMC4188433

[16] Luke B Brown MB Wantman E Stern JE Baker VL Widra E . Application of a validated prediction model for *in vitro* fertilization: comparison of live birth rates and multiple birth rates with 1 embryo transferred over 2 cycles vs 2 embryos in 1 cycle. Am J Obstetrics Gynecology. (2015) 212:676.e1–7. doi: 10.1016/j.ajog.2015.02.005 25683965 PMC4416976

[17] Gardner DK Lane M Stevens J Schlenker T Schoolcraft WB . Blastocyst score affects implantation and pregnancy outcome: towards a single blastocyst transfer. Fertility Sterility. (2000) 73:1155–8. doi: 10.1016/s0015-0282(00)00518-5 10856474

[18] Indrayan A Mishra S . Assessing the adequacy of a prediction model. Indian J Community Med Off Publ Indian Assoc Prev Soc Med. (2025) 50:739–44. doi: 10.4103/ijcm.ijcm_567_24 41017898 PMC12470336

[19] Van Calster B Wynants L Verbeek JFM Verbakel JY Christodoulou E Vickers AJ . Reporting and interpreting decision curve analysis: a guide for investigators. Eur Urol. (2018) 74:796–804. doi: 10.1016/j.eururo.2018.08.038 30241973 PMC6261531

[20] Onishi K Inoue D Kida Y Kojima M Fukunaga N Asada Y . Transfer strategy minimizing twin risk of double blastocyst transfers based on prediction models. Reprod Med Biol. (2026) 25:e70064. doi: 10.1002/rmb2.70064 42253902 PMC13239488

[21] Zhang Q Wang X Zhang Y Lu H Yu Y . Nomogram prediction for the prediction of clinical pregnancy in freeze-thawed embryo transfer. BMC Pregnancy Childbirth. (2022) 22:629. doi: 10.1186/s12884-022-04958-8 35941542 PMC9361510

[22] Cedars MI . Evaluation of female fertility-AMH and ovarian reserve testing. J Clin Endocrinol Metab. (2022) 107:1510–9. doi: 10.1210/clinem/dgac039 35100616

[23] te Velde ER Pearson PL . The variability of female reproductive ageing. Hum Reprod Update. (2002) 8:141–54. doi: 10.1093/humupd/8.2.141 12099629

[24] Halvaei I Litzky J Esfandiari N . Advanced paternal age: effects on sperm parameters, assisted reproduction outcomes and offspring health. Reprod Biol Endocrinol RB&E. (2020) 18:110. doi: 10.1186/s12958-020-00668-y 33183337 PMC7664076

[25] Dain L Auslander R Dirnfeld M . The effect of paternal age on assisted reproduction outcome. Fertility Sterility. (2011) 95:1–8. doi: 10.1016/j.fertnstert.2010.08.029 20932518

[26] Kasius A Smit JG Torrance HL Eijkemans MJ Mol BW Opmeer BC . Endometrial thickness and pregnancy rates after IVF: a systematic review and meta-analysis. Hum Reprod Update. (2014) 20:530–41. doi: 10.1093/humupd/dmu011 24664156

[27] Mahutte N Hartman M Meng L Lanes A Luo ZC Liu KE . Optimal endometrial thickness in fresh and frozen-thaw *in vitro* fertilization cycles: an analysis of live birth rates from 96,000 autologous embryo transfers. Fertility Sterility. (2022) 117:792–800. doi: 10.1097/01.ogx.0000856324.89575.d1 35109980

[28] Glujovsky D Farquhar C Quinteiro Retamar AM Alvarez Sedo CR Blake D . Cleavage stage versus blastocyst stage embryo transfer in assisted reproductive technology. Cochrane Database Systematic Rev. (2016) 30:Cd002118. doi: 10.1002/14651858.cd002118.pub4 27357126

[29] Jin W Lin J Wang P Yang H Jin C . Screening the predictors for live birth failure in women after the first frozen embryo transfer based on the Lasso algorithm: a retrospective study. Zygote (Cambridge England). (2023) 31:350–8. doi: 10.1017/s0967199423000217 37183670

[30] Schouten N Wang R Torrance H Van Tilborg T Bastu E Bergh C . Development and validation of a gonadotropin dose selection model for optimized ovarian stimulation in IVF/ICSI: an individual participant data meta-analysis. Hum Reprod Update. (2025) 31:116–32. doi: 10.1093/humupd/dmae032 39707165 PMC11879166

[31] Ratna MB Bhattacharya S Abdulrahim B McLernon DJ . A systematic review of the quality of clinical prediction models in *in vitro* fertilisation. Hum Reprod (Oxford England). (2020) 35:100–16. doi: 10.1093/humrep/dez258 31960915

